# CD18 Antibody Application Blocks Unwanted Off-Target T Cell Activation Caused by Bispecific Antibodies

**DOI:** 10.3390/cancers13184596

**Published:** 2021-09-13

**Authors:** Joseph Kauer, Fabian Vogt, Ilona Hagelstein, Sebastian Hörner, Melanie Märklin, Stefanie Maurer, Helmut R. Salih, Gundram Jung, Latifa Zekri

**Affiliations:** 1 German Cancer Consortium (DKTK) and German Cancer Research Center (DKFZ) Partner Site Tübingen, Department of Immunology, Interfaculty Institute for Cell Biology, University of Tübingen, 72076 Tübingen, Germany; fabianvogt@gmx.net (F.V.); s.hoerner@dkfz-heidelberg.de (S.H.); gundram.jung@uni-tuebingen.de (G.J.); l.zekri-metref@dkfz-heidelberg.de (L.Z.); 2 Clinical Collaboration Unit Translational Immunology, German Cancer Consortium (DKTK), Department of Internal Medicine, University Hospital Tübingen, 72076 Tübingen, Germany; Ilona.hagelstein@med.uni-tuebingen.de (I.H.); melanie.maerklin@med.uni-tuebingen.de (M.M.); stefanie.maurer@med.uni-tuebingen.de (S.M.); helmut.salih@med.uni-tuebingen.de (H.R.S.); 3 Department of Oncology and Hematology, University Clinic Heidelberg, 69118 Heidelberg, Germany; 4 DFG Cluster of Excellence 2180 ‘Image-Guided and Functional Instructed Tumor Therapy’ (iFIT), Eberhard Karls University, 72076 Tübingen, Germany; 5 Department of Radiology, Memorial Sloan Kettering Cancer Center, New York, NY 10065, USA

**Keywords:** bispecific antibodies, cytokine release syndrome, CD18, immunotherapy, T cell activation

## Abstract

**Simple Summary:**

Bispecific antibodies are a very effective immunotherapy against different types of cancer since they activate T cells in the presence of tumor cells. However, they can cause severe side effects, such as a systemic inflammation called cytokine release syndrome. We aimed to clarify an important mechanism that causes cytokine release syndrome. In cocultures of T cells with endothelial cells or lymphoid cells, application of bispecific antibodies can induce T cell activation and cytokine release in the absence of tumor cells. By blocking the adhesion molecule CD18, this interaction is interrupted and the unwanted T cell activation is diminished. CD18 blockade, however, does not interfere with T cell activation when tumor cells are present. Therefore, CD18 blockade could prevent side effects of bispecific antibodies without decreasing the anti-tumor effect.

**Abstract:**

T cell-recruiting bispecific antibodies (bsAbs) are successfully used for the treatment of cancer. However, effective treatment with bsAbs is so far hampered by severe side effects, i.e., potentially life-threatening cytokine release syndrome. Off-target T cell activation due to binding of bispecific CD3 antibodies to T cells in the absence of target cells may contribute to excessive cytokine release. We report here, in an in vitro setting, that off-target T cell activation is induced by bsAbs with high CD3 binding affinity and increased by endothelial- or lymphoid cells that act as stimulating bystander cells. Blocking antibodies directed against the adhesion molecules CD18/CD54 or CD2/CD58 markedly reduced this type of off-target T cell activation. CD18 blockade—in contrast to CD2—did not affect the therapeutic activity of various bsAbs. Since CD18 antibodies have been shown to be safely applicable in patients, blockade of this integrin holds promise as a potential target for the prevention of unwanted off-target T cell activation and allows the application of truly effective bsAb doses.

## 1. Introduction

Bispecific antibodies (bsAbs) consisting of a binding site targeting a tumor-associated antigen (TAA) and the TCR-associated signaling molecule CD3 can redirect T cells against tumor cells. In particular, when Fc-depleted to prevent binding to FcγR+ cells, bsAbs supposedly act in a target-cell restricted manner, as they should induce T cell activation only after binding both, the TAA and CD3 [1,2,3,4,5]. So far, bsAbs have been successfully used for the treatment of B cell derived-malignancies. However, their application can result in the development of a potentially lethal cytokine release syndrome (CRS) [6,7], which limits safely applicable doses. In the case of blinatumomab, the benchmark CD19xCD3 bsAb, daily doses are less than 50 µg per patient [6]. This dose limitation, which can also be observed with other bsAbs, [8,9] is possibly due to off-target T cell activation and can be induced by two phenomena: (1) The TAA is not tumor-specific, thus resulting in bsAb-mediated “on-target off-tumor” T cell activation due to the presence of normal antigen-expressing cells. Blinatumomab certainly faces this problem, since its target antigen CD19 is expressed on healthy B cells. (2) True “off-target” activation, reflected by the induction of T cell activation by the CD3 part of the bsAb in the absence of the target antigen. We here report that at high bsAb concentrations true off-target activation may occur and that endothelial and lymphoid cells can act as stimulating bystander cells (SBCs) by promoting T cell activation in the absence of target antigen as revealed by analyses involving various TAAxCD3 bsAbs. In this context, we identified CD11a/CD18 (LFA-1) as an important mediator of this type of off-target T cell activation.

## 2. Materials and Methods

### 2.1. Cells and Reagents

Heparinized blood was obtained from healthy male and female donors (approved by the ethics committee of the University Hospital Tübingen, authorization 156/2012BO1) and transported at room temperature. Peripheral blood mononuclear cells (PBMCs) were isolated by density gradient centrifugation using Biocoll Cell Separation Solution (Biochrom, Berlin, Germany). The mean processing time was 120 min, ranging from 100 to 150 min. PBMCs were kept in RPMI 1640 medium until use for no longer than 24 h. No frozen PBMCs were utilized. Viability of >95% was confirmed by staining with Trypan Blue and Turk’s solution (both from Sigma-Aldrich, St. Louis, MS, USA) The cell lines 22Rv1, C1R, Daudi, JY, Nalm-16, Raji, and SKW6.4 were obtained from the German Collection of Microorganisms and Cell Cultures (DSMZ, Braunschweig, Germany). Cell lines were repeatedly tested negative for mycoplasma. Human umbilical vein endothelial cells (HUVECs) were purchased from Promocell (Heidelberg, Germany) and kept in Endothelial Cell Growth Medium (Promocell). PBMCs and cell lines were kept in RPMI 1640 supplemented as described earlier [10]. These studies were conducted in a laboratory that operates under exploratory research principle by using established laboratory protocols and performing general research investigative assays. The T cell assays performed in this work comply with the MIATA recommendations for reporting such methods.

### 2.2. Antibodies and Flow Cytometry

The recombinant bsAb NP-CU (PSMAxCD3), N19-CU (CD19xCD3) and NM-CU (CSPG4xCD3) were generated at our institution in the Fabsc or IgGsc format as described previously [10,11]. In brief, the CD3 binding site comprises a single chain derived from the CD3 antibody UCHT-1 or OKT-3. PSMA, CD19, and CSPG4 binding sites are derived from the antibodies J591, 4G7, and 9.2.27, respectively. All proteins are subjected to analytical and preparative size exclusion chromatography using Superdex S200-Increase 10/300GL and HiLoad 16/60 columns (GE Healthcare, Chicago, IL, USA), respectively, and only the fractions containing the monomeric form were used. Fc receptor binding and complement fixation were attenuated by introducing the following mutations and substitution in the CH2 domain: E233P; L234V; L235A; ΔG236; D265G; A327Q; A330S (EU-index). The presence of endotoxins or aggregates was ruled out after production.

Blocking antibodies against CD2 (clone RPA-2.10), CD11a (HI111), CD11b (ICRF44), CD18 (TS1/18), CD40 (5C3), CD54 (HA58 and HCD54), CD58 (TS2/9), CD62E (HAE-1f), CD62L (DREG-56), CD80 (2D10), CD86 (BU63), CD102 (CBR-1C2/2), CD137L (TKS-1), and CD252 (11C3.1) were purchased from BioLegend (San Diego, CA, USA).

Fluorochrome-labeled antibodies directed against CD4, CD8, CD11a, CD69 and the respective isotype control antibodies were purchased from BioLegend. CD45-AmCyan was purchased from BD Biosciences (Franklin Lakes, NJ, USA).

For flow cytometry-based assays, 50,000 PSMA+ 22Rv1 cells or 100,000 CD19+ Nalm-16 cells were incubated in 96 well plates together with 100,000 PBMCs, bsAb at 1 μg/mL and blocking reagents at 10 μg/mL. PBMCs only, together with target cells and PBMCs+ phytohemagglutinin L (PHA, 10 µg/mL) were used as internal controls. To investigate the effect of CD19xCD3 in the off-target T cell activation, B cell-depleted PBMC were used. B cell depletion was performed using CD20 MicroBeads (Miltenyi Biotec, Bergisch Gladbach, Germany) and was confirmed by flow cytometry. After 3 days, flow cytometric analysis was performed. For the competition and antigen shift assays, on-target activated cells were additionally incubated with titrated CD11a or CD18 for 1h at 4 °C and 30h at 37 °C, respectively. Directly labeled CD11a and CD18 were added to the cells at a saturating amount (10 µg/mL) and incubated for an additional hour at 4 °C. The results were obtained by flow cytometry.

For all experiments, at least 100,000 events were recorded for each sample. The gating strategy was as follows: Mononuclear cells (FSC-H vs. SSC-H), singlets (FSC-A vs. FSC-H), viable (7-AAD-), CD45+ leukocytes, CD4+ or CD8+ T cells, activated T cells CD69+ (Figure S5). Fc receptor binding to antibodies was blocked by Flebogamma DIF (Grifols, Barcelona, Spain) at 50 μg/mL. Data were acquired using a FACSCanto II or a FACSCalibur (BD Biosciences, Franklin Lakes, NJ, USA). Flow cytometry data were analyzed using FlowJo_V10 (Tree Star, Ashland, OR, USA). Specific fluorescence index values (SFIs) were obtained by division of median fluorescence indices measured with the antigen-specific mAb by median fluorescence exerted by the isotype control antibody. Antigen expression was considered as positive in the case of SFI ≥ 1.5, our predefined cut-off. During a T cell proliferation assay, 100,000 PBMCs from healthy donors and irradiated (100 Gy) target cells or SBCs (E:T ratio 1:1) were seeded in triplicates in 96 well plates and incubated with bsAb (1 μg/mL). When indicated, cell culture plates were coated overnight at 4 °C with 5 µg/mL of His-tagged ICAM-1 (ACROBiosystem, Newark, DE, USA). After 48 h, cells were pulsed with 3H-methyl thymidine (0.5 μCi/well) and incubated for another 20 h until harvesting on filter mats. Radioactivity uptake was determined using a MicroBeta2 2450 Microplate liquid scintillation counter (PerkinElmer, Waltham, MA, USA).

### 2.3. Real-Time Tumor Cell Killing Assay

Real-time lysis of PSMA+ tumor cells was assessed by xCELLigenceTM assays. Adherent 22Rv1 cells (30,000 cells/well) were applied to a 96 well gold-coated E-plate and incubated for 20 h. After constitution of cell indices > 1.5, indices were normalized to 1.0. After that, PBMCs (100,000 cells/well) and a PSMAxCD3 bsAb at 1 μg/mL together with the respective blocking antibodies were added and cell indices were measured every 15 min to determine the number of viable tumor cells. The Kill-Time-50 (KT50) was defined as time span after PBMC addition and eradication of 50% of 22Rv1 tumor cells.

### 2.4. Legendplex Cytokine Arrays

LEGENDplex cytokine arrays (Human TH1, 5-plex, BioLegend, San Diego, CA, USA) were performed according to the manufacturer’s instruction using supernatants from flow cytometry-based kill assays.

### 2.5. Statistical Analysis

Data are displayed as mean ± standard deviation or as boxplots with min/max whiskers. For statistical analysis, Graphpad_V10 was utilized. Mann–Whitney U tests or unpaired *t*-tests were used to test for significance in unpaired data sets.

## 3. Results

### 3.1. Off-Target T Cell Activation upon bsAb Binding in the Absence of Target Cells

**Figure 1:**
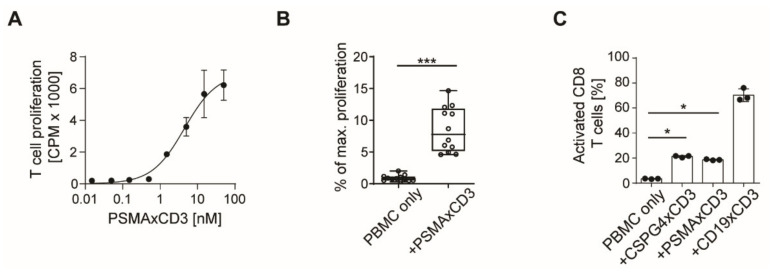

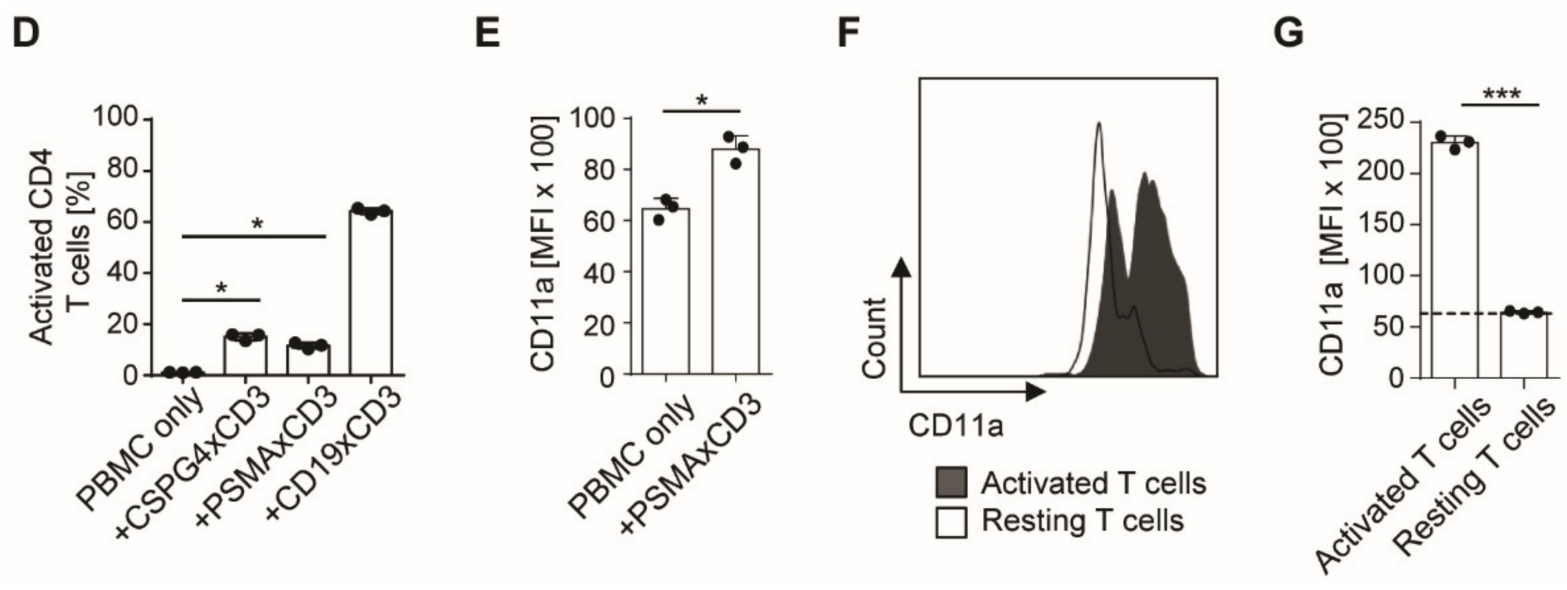
Off-target T cell activation by bsAbs in the absence of target cells. (**A**) Off-target T cell proliferation was measured in a 3 day 3H-thymidine incorporation assay with a PSMAxCD3 bsAb and 100,000 PBMC. Exemplary results from experiments using PBMC from 5 different donors (mean ± SD of triplicates). (**B**) T cell proliferation in a thymidine assay with PBMCs from 12 donors using PSMAxCD3 bsAb at 5 nM (boxplots with min/max whiskers, unpaired *t*-test). Standardization to maximum proliferation (set to 100%) as observed with PBMC + phytohemagglutinin was performed. (**C**,**D**) Flow cytometric analysis of T cell activation (CD69 expression) on CD8+ and CD4+ T cells after 3-day stimulation of 100,000 PBMC with different bsAb at 5 nM (*n* = 3, mean ± SD, unpaired *t*-test). (**E**) Flow cytometric analysis of CD11a expression on CD8+ T cells upon 3 day off-target stimulation with PSMAxCD3 bsAb at 5 nM (*n* = 3, mean± SD, unpaired *t*-test). (**F**,**G**) Median CD11a fluorescence intensity on off-target activated CD8+CD69+ T cells and resting CD8+CD69- T cells after 3 days stimulation with a PSMAxCD3 antibody as measured by flow cytometry (*n* = 3, mean ± SD, unpaired *t*-test). Open peaks indicate resting T cells, shaded peaks show activated CD69+ T cells. Dotted line indicates expression level on unstimulated PBMC. * *p* < 0.05, *** *p* < 0.001.

As PSMA is not expressed on any lineage within PBMC cultures [12], we incubated PBMC from healthy donors with a PSMAxCD3 antibody in the Fabsc format [10] to evaluate off-target T cell activation. A 3-day ^3^H-thymidine incorporation assay showed proliferation of T cells in the absence of target cells (Figure 1A) at concentrations > 1 nM. Combined analyses with PBMC of 12 donors revealed in average 8% off-target T cell activation compared to the maximum proliferation induced by phytohemagglutinin (PHA, 10 µg/mL) (Figure 1B). Off-target activation was observed not only with PSMAxCD3, but also with CSPG4xCD3-bsAb, the latter targeting chondroitin sulfate proteoglycan (CSPG), not present in PBMCs [12,13,14]. CD19xCD3 was included as a positive control to monitor the on-target T cell activation mediated by CD19 positive B cells within PBMC preparations. Off-target T cell activation was accompanied by expression of the activation marker CD69 on both CD8+ and CD4+ T cells (Figure 1C,D). Since T cell activation results in increased expression of integrins and therefore increased adhesion, we analyzed the off-target activated T cells by flow cytometry and observed upregulation of the integrin CD11a/CD18 (LFA-1) (Figure 1E). The expression could be attributed to a subset of activated T cells, whereas resting CD69- T cells did not exhibit CD11a upregulation (Figure 1F,G). To test whether the off-target T cell activation correlates with the CD3 binding affinity, we compared the activity of the Fabsc antibody with that of an IgGsc molecule comprising the same CD3 single chain. However due to spatial restrictions the affinity of the CD3 moiety in the IgGsc molecule is considerably lower and in contrast to the Fabsc molecule [11], this antibody showed no off-target T cell activation (Figure S1). The latter also holds true for a Fabsc molecule containing a low-affinity single-chain CD3 binder, indicating that regardless of CD3 binding valency and the bsAb format, only high CD3 binding affinity can contribute to true off-target activation.

### 3.2. Endothelial and Lymphoid Cells Enhance Off-Target T Cell Activation by Acting as Stimulating Bystanders

**Figure 2:**
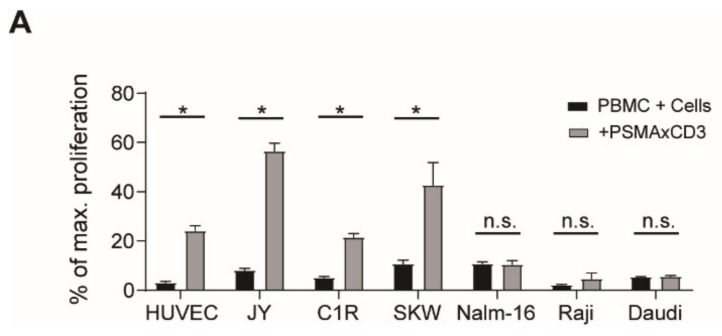

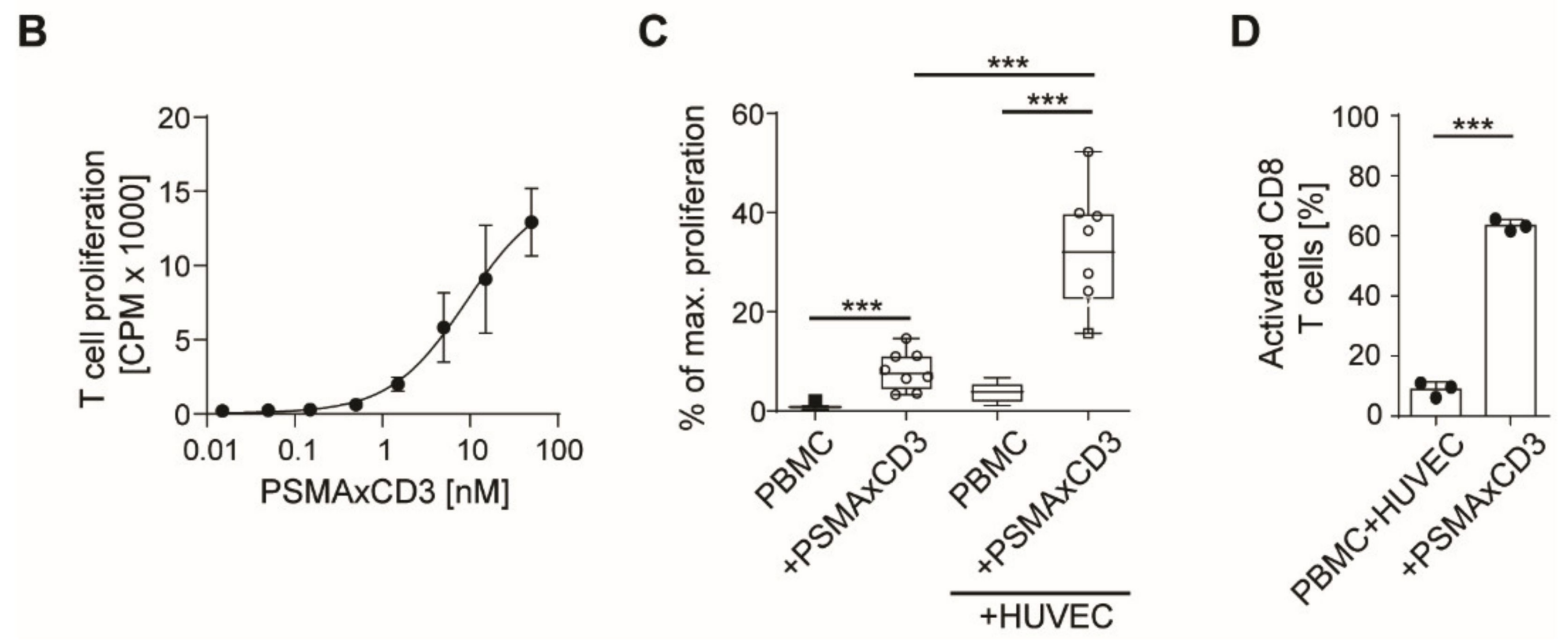
Increased off-target activation in the presence of stimulating bystander cells. (**A**) Off-target T cell proliferation was measured using 100,000 PBMCs, PSMAxCD3 bsAb at 5 nM and different cell lines (50,000–100,000/well) in a 3 day 3H-thymidine incorporation assay (*n* = 3, mean ± SD, unpaired *t*-test). (**B**) T cell proliferation in a thymidine assay with 50,000 HUVEC and 100,000 PBMCs and ascending doses of PSMAxCD3 bsAb. Representative results from experiments using PBMC from *n* = 7 different donors. Standardization to maximum proliferation (set to 100%) as observed with PBMC + phytohemagglutinin was performed. (**C**) T cell proliferation in a thymidine assay using HUVEC, PBMC and PSMAxCD3 at 5 nM (*n* = 7, boxplots with min/max whiskers, unpaired *t*-test). Standardization to maximum proliferation (set to 100%) as observed with PBMC + phytohemagglutinin was performed. (**D**) Flow cytometric analysis of Activation (CD69 expression) of CD8 T cells after 3 day off-target stimulation with 50,000 HUVECs and PSMAxCD3 bsAb at 5 nM (*n* = 3, mean ± SD, unpaired *t*-test). * *p* < 0.05, *** *p* < 0.001.

Besides lymphocytes, endothelial cells are among the first cell populations encountered by circulating bsAb coated T cells after therapeutic application but are rarely included in in vitro models studying CRS. To evaluate the impact of these cells, we tested whether off-target T cell activation, as described above, is influenced by the presence of endothelial cells (using HUVEC) or lymphoid cell lines. These cells do not express the target antigen PSMA (Figure S2). Interestingly, T cell proliferation analyses revealed that HUVECs, as well as some lymphoid cell lines (JY, C1R and SKW6.4) enhanced off-target T cell activation (Figure 2A), and were thus designated as stimulating bystander cells (SBCs). Other lymphoid cell lines, such as the B cell lines Nalm-16, Raji, and Daudi, did not exhibit stimulatory properties. SBC-mediated off-target activation was observed at bsAb concentrations > 1 nM (Figure 2B), a concentration that may be reached at least if higher doses of bsAb are applied [15,16]. Using PBMC from seven healthy donors, we found that 30% of the maximum-inducible proliferation was observed due to the presence of SBCs (Figure 2C) and mirrored by increased CD69 expression on T cells (Figure 2D).

### 3.3. Integrin Ligands Are Involved in BsAb-mediated Off-Target T Cell Activation

Selectins, such as CD62E, and integrin ligands, such as CD54 (ICAM-1) and CD102 (ICAM-2), are expressed on endothelial cells and are important mediators of leukocyte adhesion. To unravel the functional basis of the stimulating properties of SBC, we analyzed the expression of different costimulatory (CD40, CD80, CD86, CD137L, CD252) and adhesion molecules (CD54, CD58, CD62E, CD62L, CD102) by flow cytometry on two SBCs (HUVEC and SKW6.4), as well as a non-stimulating cell line (Nalm-16).

**Figure 3:**
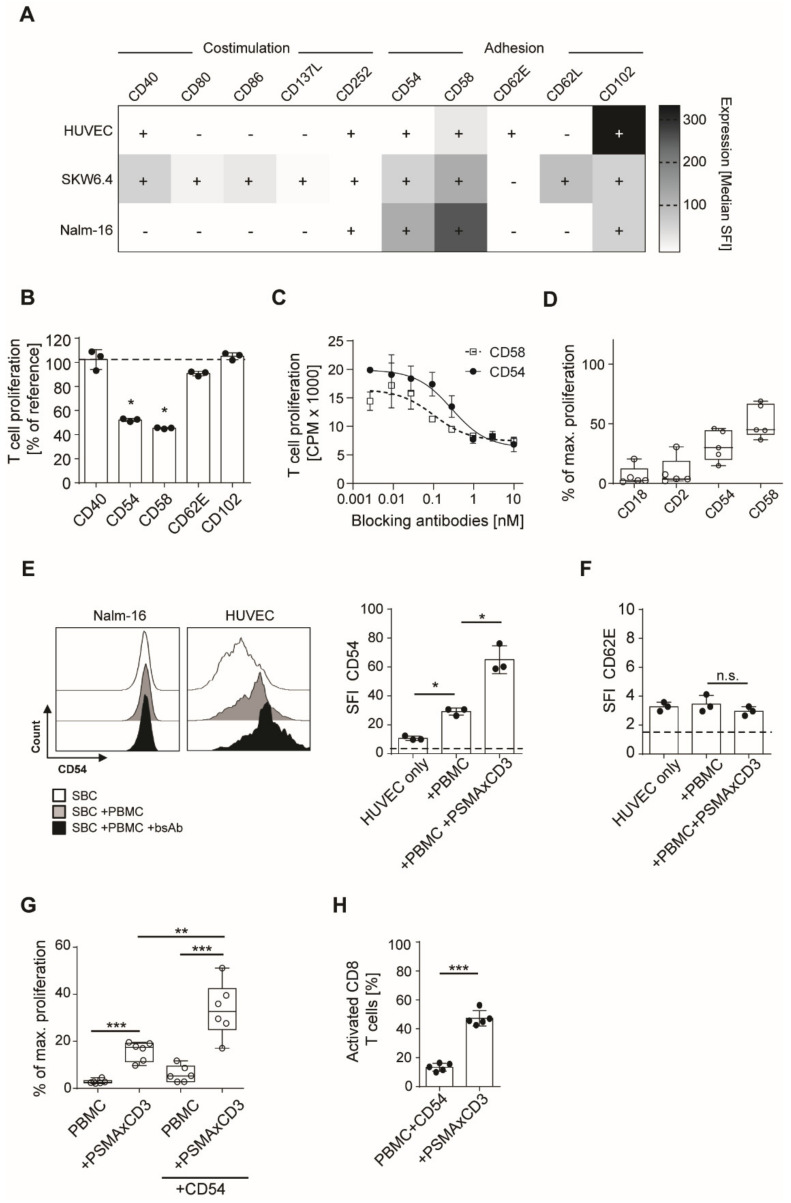
Expression and blockade of costimulatory molecules on stimulating bystander cells (SBC). (**A**) Expression of different costimulatory and adhesion molecules was analyzed on different cell lines by flow cytometry. Specific fluorescence intensities (SFI) are depicted. (**B**) Off-target T cell activation was induced in a 3 day 3H-thymidine incorporation assay (*n* = 3) using 100,000 PBMC, 30,000 HUVEC and a PSMAxCD3 antibody at 1 µg/mL. Addition of different blocking antibodies at 10 µg/mL directed against costimulatory/adhesion molecules on SBCs (mean ± SD, unpaired *t*-test). (**C**) Inhibition of off-target activation by CD54 and CD58 antibodies in a thymidine assay. (**D**) Inhibition of off-target activation by different blocking antibodies in thymidine assays (*n* = 5, mean ± SD, Mann–Whitney U test). Standardization to maximum proliferation (set to 100%) as observed with PBMC + phytohemagglutinin was performed. (**E**) Flow cytometric analysis of CD54 expression on HUVEC and Nalm-16 upon 24 h stimulation with PBMC or PBMC (*n* = 4) and PSMAxCD3 antibody (mean ± SD, Mann–Whitney U test). (**F**) Flow cytometric analysis of CD62E expression on HUVEC upon stimulation as described in E (*n* = 4, mean ± SD, unpaired *t*-test). Dotted line indicates the predefined cut-off of SFI 1.5. (**G**) Thymidine assays were performed in the presence of coated CD54 protein, PBMC and PSMAxCD3 at 5 nM (*n* = 6, boxplots with min/max whiskers, unpaired *t*-test). Standardization to maximum proliferation (set to 100%) as observed with PBMC + phytohemagglutinin was performed. (**H**) Analysis of CD69 expression on CD8 T cells after 3 days off-target stimulation with coated CD54 protein and 5 nM of PSMAxCD3 bsAb by flow cytometry. Statistical analyses were performed using the unpaired *t*-test (*n* = 6, mean ± SD). * *p* < 0.05, ** *p* < 0.01, *** *p* < 0.001.

CD40 was identified as the only molecule present on the stimulating cell lines HUVEC and SKW6.4 but not on Nalm-16 cells that lacked SBC activity (Figure 3A). In contrast, three integrin-ligands (CD54, CD58 and CD102) were expressed on SBCs, but also on non-stimulatory cell lines. To determine which costimulatory or adhesion molecules are involved in the off-target T cell activation, we incubated off-target activated PBMC with blocking antibodies directed against the identified molecules. Despite its presence on SBCs, blockade of CD40 did not reduce off-target activation (Figure 3B). However, blockade of CD54 and CD58 resulted in a potent reduction of off-target stimulation (Figure 3C,D). Antibodies directed against CD102 had no notable effect. Those results are in accordance with the fact that CD54 is massively upregulated on activated endothelium, in contrast to CD102, which mostly mediates extravasation of immune cells through non-activated endothelium [17]. Indeed, we found that CD54 was moderately expressed on resting HUVECs, but greatly increased upon off-target stimulation (Figure 3E). In contrast, CD54 expression on the non-stimulating cell line (Nalm-16) remained unchanged (Figure 3E). No induction of CD62E expression was detectable on HUVECs during off-target stimulation (Figure 3F). To investigate in a more direct way the capacity of CD54 to enhance off-target T cell activation, a proliferation assay was performed in the presence of coated CD54 protein. Indeed, 30% of the maximum inducible proliferation, accompanied by an increase in CD69 expression on T cells was observed in the presence of the CD54 protein (Figure 3G,H). Overall, our results suggest that integrins rather than selectins play a major role in off-target T cell activation and that CD54 triggers this effect.

### 3.4. BsAb-mediated Off-Target T Cell Activation Is Prevented by Integrin Blockade

**Figure 4:**
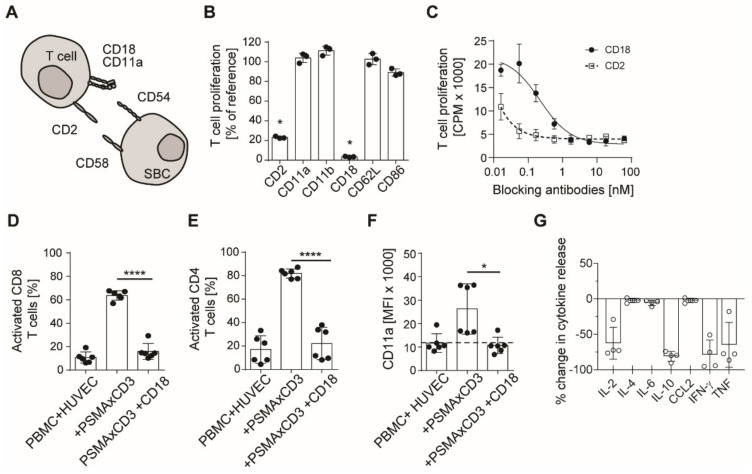

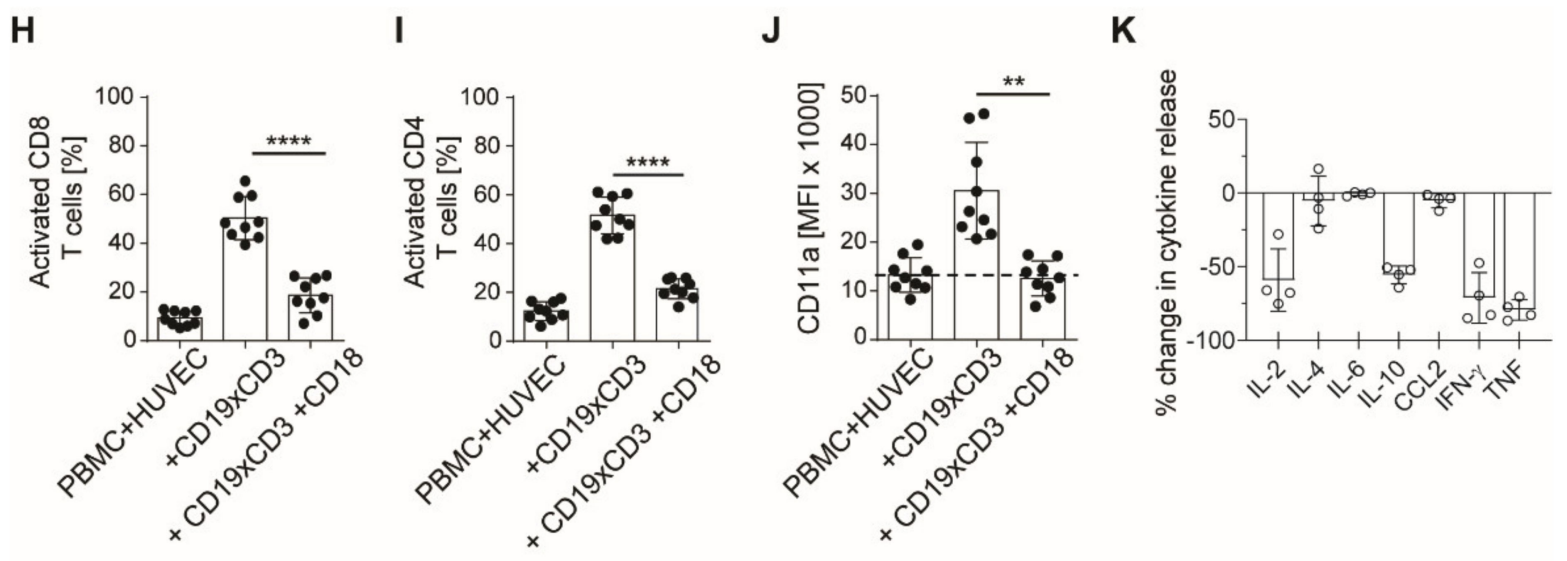
Reduced off-target activation by blockade of adhesion molecules on T cells. (**A**) Schematic illustration of costimulation by interaction of CD2 and CD11a/CD18 with CD58 and CD54, respectively. (**B**) Off-target T cell activation was induced in a 3 day 3H-thymidine incorporation assay (*n* = 3, mean ± SD, unpaired *t*-test) using 100,000 PBMC, 30,000 HUVEC and a PSMAxCD3 antibody at 1 µg/mL. Addition of different blocking antibodies at 10 µg/mL directed against costimulatory/adhesion molecules on T cells. (**C**) Inhibition of off-target T cell proliferation by CD18 and CD2 antibodies in a thymidine assay as described in **B** (*n* = 3, mean ± SD). (**D,E**) Flow cytometric analysis of CD69 expression on CD8+ and CD4+ T cells upon off-target stimulation with 30,000 HUVEC and a PSMAxCD3 antibody at 1 µg/mL (*n* = 6, mean ± SD, unpaired t test). (**F**) Flow cytometric analysis of CD11a expression on CD8+ T cells through off-target activation as described in d (*n* = 6). (**G**) Cytokine release analyzed by LEGENDplex (*n* = 4) during off-target activation induced by PSMAxCD3 antibody utilizing supernatants from assays as described in **D**,**E**. (**H**,**I**) Off-target stimulation of CD19xCD3 in the presence of 30,000 HUVEC, CD19xCD3 antibody at 1 µg/mL and B cell-depleted PBMC (*n* = 6). The blocking CD18 antibody was added at 10 µg/mL. Analysis of CD69 expression on CD8+ and CD4+ T cells was obtained by flow cytometry. (**J**) Flow cytometric analysis of CD11a expression on CD8+ T cells through off-target activation as described in G (*n* = 9). (**K**) Cytokine release analyzed by LEGENDplex (*n* = 4) during off-target activation induced by CD19xCD3 antibody utilizing supernatants from assays as described in H-I. * *p* < 0.05, ** *p* < 0.01, **** *p* < 0.0001.

To functionally characterize T cell associated molecules that mediate T cell co-stimulation and adhesion (Figure 4A) and to further confirm the functional relevance of integrins in off-target T cell activation, we incubated off-target activated PBMC with blocking antibodies directed against different costimulatory and adhesion molecules expressed by T cells. Maximum reduction of bsAb-induced off-target T cell proliferation was observed by blocking CD2 and CD18 (integrin β2), a subunit of LFA-1 (Figure 4B). Maximum blocking effects were observed at concentrations of approximately 1 nM (Figure 4C). The effect of CD18 blockade observed with regard to T cell proliferation was mirrored by markedly reduced CD69 expression on off-target stimulated CD8+ and CD4+ T cells (Figure 4E,F,H,I). Furthermore, CD11a expression, as observed during unspecific activation in the presence of HUVECs, was also decreased in the presence of CD18 blocking antibodies (Figure 4F,J). This holds true for cytokines that were also significantly reduced in the presence of the CD18 antibody (Figure 4G,K). Results were confirmed in experiments utilizing SKW6.4 cells as different SBC (Figure S3).

### 3.5. Blockade of CD2 and CD18 Differently Affects On-Target T Cell Activation

**Figure 5:**
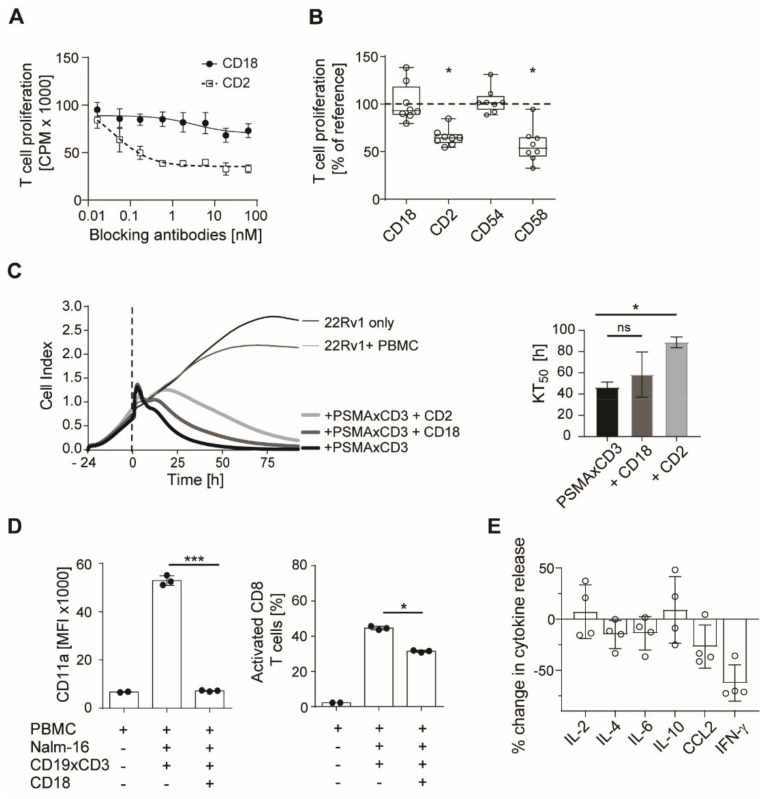
Effects of blocking antibodies on on-target activation. (**A**) On-target T cell activation was measured in a 3 day 3H-thymidine incorporation assay using 100,000 PBMC, 100,000 CD19+ Nalm-16 cells and a CD19xCD3 antibody at 5 nM and ascending doses of CD2 and CD18 blocking antibodies. Representative results from experiments with three donors (mean ± SD from triplicates). (**B**) On-target activation as described in A (*n* = 8, boxplots with min/max whiskers, unpaired *t*-test). Activation with 100,000 PBMC, 100,000 CD19+ Nalm-16 cells and a CD19xCD3 antibody at 5 nM was set to 100%. (**C**) Real-time tumor cell lysis using xCELLigence. 24 h after addition of PSMA+ 22Rv1 cells, PBMCs and PSMAxCD3 antibodies at 1 µg/mL were added. Cell indices correspond to the number of viable 22Rv1 tumor cells. The graph represents the mean of three independent experiments. A Killing Time (KT50) corresponding to the time required to kill 50% of 22Rv1 target cells are presented in the table. ND. not detected. (**D**) Flow cytometric analysis of CD11a and CD69 expression upon on-target stimulation as described in **A** (**n** = 3, mean ± SD, unpaired *t*-test). (**E**) Cytokine release during on-target activation analyzed by LEGENDplex analysis (*n* = 4, bars depicting mean ± SD, Mann–Whitney U test) utilizing supernatants from assays as described in **A**. * *p* < 0.05, *** *p* < 0.001.

Next, we studied how blocking antibodies against CD2 and CD18 affected desired on-target T cell activation induced by bsAbs. Using thymidine incorporation assays, we found that CD2 antibodies inhibited T cell activation in the presence of CD19 positive Nalm-16 cells and a CD19xCD3 antibody already at doses < 1 nM by about 50%. On the contrary, CD18 blockade did not affect therapeutically desired T cell activity (Figure 5A,B). CD18 blockade of tumor cell killing was further evaluated using real-time impedance-based xCELLigence assays to study potential effects on target cell killing. While PSMAxCD3 potently reduced the number of viable target cells, only a marginal delay in bsAb-induced killing of 22Rv1 prostate cancer cells was observed in the presence of the CD18 blocking antibody (Figure 5C). In contrast, killing time was significantly longer in the presence of the CD2 antibody. This observation was confirmed by calculating the KT50, corresponding to the time required to kill 50% of 22Rv1 target cells. Results obtained in a flow cytometry-based kill assay with CD19+ target cells and a CD19xCD3 bsAb show that neither CD18 blockade nor combined blockade with CD54 antibodies (at 1 µg/mL) and CD18 antibodies (at 0.1 µg/mL or 0.01µg/mL) show any negative impact on tumor cell killing (Figure S6). Surprisingly, however, CD18 blockade greatly reduced the expression of CD11a on T cells during on-target stimulation, but only showed moderate effects on activation levels as revealed by analysis of CD69 expression (Figure 5F). In subsequent experiments we found that this effect is due to an internalization of the LFA-1 complex upon the presence of a CD18 antibody (Figure S4). Furthermore, IFNγ release was reduced in the on-target experimental setting in the presence of CD18 antibodies (Figure 5G).

Together, these data demonstrate that CD18 blockade can serve to reduce undesired off-target T cell activation induced by SBCs without hampering on-target activation.

## 4. Discussion

BsAbs can activate T cells against malignant cells. If binding to Fc-receptor positive cells is prevented, T cell activation should occur only in the presence of the tumor target antigen cells to which the bsAb bind. However, the rapid occurrence of cytokine release after application of bsAbs suggests that they may exert some T cell activation in the absence of target cells. In this work, we observed this type of off-target T cell activation utilizing different bsAbs in the Fabsc-format comprising a high affinity CD3 binding moiety. These data were obtained using bsAbs directed against different antigens that are not expressed on human PBMCs, such as PSMA and CSPG4 [12,13,14]. Furthermore, we established an experimental setting that allows to study off-target T cell activation in the presence of endothelial and lymphoid cells serving as stimulating bystander cells. Such cells are present in high abundance and are among the first cells to be encountered by T cells after application of bsAbs. We found that blockade of different costimulatory and adhesion molecules, such as CD11a/CD18 and CD2 effectively blocks unwanted off-target T cell activation. Whereas our findings provide important proof of principle, confirmation in suitable animal models in future studies is warranted, particularly prior to translation of our findings to clinical evaluation in humans. In particular, homing of T cells to the tissue during bispecific antibody treatment should be investigated. Indeed, inherited CD18 protein defects lead to leukocyte adhesion deficiency in patients, resulting in severe bacterial and fungal infection [18,19]. Inhibition of T cell trafficking to tissues by CD18 blockade could hamper therapeutic efficacy of bsAb [20]. Therefore, CD18 blockade should be limited to a short period of time.

Molema et al. described the activation of endothelial cells and subsequent adhesion of T cells after on-target activation by bsAbs [21]. In accordance with the latter, we also observed upregulation of adhesion molecules upon bsAb induced activation, even in the absence of target cells. Endothelial cells and several B cell lines were identified as capable of acting as SBCs that amplify off-target activation. Since SBC activity was found to be limited to certain cell lines, we concluded that this capability may be mediated by defined costimulatory molecules. Our results suggest that not only monocytic cells, as reportedly involved in chimeric antigen receptor (CAR) T cell- and bsAb-mediated CRS [22,23,24,25], but also lymphoid and endothelial cells interact with T cells during bsAb therapy and cause unwanted off-target effects. As binding of CD11a/CD18 to CD54 induces mitogenic signals in T cells, we reasoned that adhesion of T cells to epithelial cells, as observed in patients after application of bsAbs, might contribute to off-target T cell activation. To unravel the involved mechanism, our functional studies revealed that blockades of CD54 and CD58 were highly effective in reducing off-target activation. The involvement of CD54 suggests that endothelial cells are activated by direct or indirect (e.g., cytokine) stimulation by T cells and upregulate CD54 as implicated by this paper. The important role of CD54 and CD58 in off-target T cell activation was further confirmed by using antibodies against their respective counterparts on T cells, CD11a/CD18 and CD2, resulting in even more potent reduction of off-target activation.

Interestingly, soluble CD18 was described by Kragstrup et al. as being an immune modulator in sepsis. Higher levels of soluble CD18 in patients correlated with improved survival due to decreased complement activation through interaction of CD18 with the complement fragment iC3b [26]. In vivo studies should focus on the role of soluble CD18 in patients undergoing bispecific antibody therapy.

Upregulation of CD11a/CD18 occurs in response to cytokines and chemokines [27] commonly produced upon CD3-induced off-target T cells activation [22,23,25,28,29]. We have shown that a high binding affinity to CD3 apparently favors this effect [11]. Initial efforts to generate CD3-containing bsAb antibodies were often biased towards using high-affinity CD3 binders, which triggered not only potent tumor cell killing, but also high levels of cytokine release. As a consequence, several companies such as Xencor (Pasadena, CA) and Macrogenics (Gaithersburg, MD) generated bsAbs with reduced CD3 binding affinity in an attempt to reduce unwanted off-target T cells activation [30,31].

When aiming for the reduction of off-target T cell activation, any intervention should not interfere with the desired T cell activation in the presence of TAA expressing tumor cells. Our data show that CD18- in contrast to CD2- does not substantially inhibit on-target activation in several assay systems. Obviously, future analyses are needed regarding the time course of CD18 upregulation upon bsAbs application in vivo and the effective CD18 blockade doses required to overcome the off-target T cell activation side effects. These analyses should also clarify whether ICAM-1 signals prime T cells and thereby lower the CD3 activation threshold or whether both signals have to be present simultaneously.

Antagonistic CD18 antibodies such as erlizumab and rovelizumab were already tested in several clinical trials in the 1990s [32,33,34,35,36,37] and a single intravenous application constituted serum concentrations > 10 μg/mL [37]. These antibodies were intended to inhibit inflammatory tissue invasion by leukocytes for treatment of multiple sclerosis, hemorrhage, stroke, or myocardial infarction, but failed to achieve the ambitious therapeutic goals [33,34,35]. However, no substantial side effects were reported [34]. In contrast to natalizumab, another integrin-targeting antibody for the treatment of multiple sclerosis, which may induce multifocal leukoencephalopathy (PML) [38,39], in patients previously exposed to polyomavirus 2, no vulnerability towards viral infections was reported after CD18 antibodies. This is even more important given the universal role of CD18 in leukocyte adhesion and migration [40].

## 5. Conclusions

In summary, CD18 antibodies may be promising candidates for the prevention of off-target induced side effects of bsAb therapy and hold promise to improve the safety and tolerability of this important immunotherapeutic approach.

## 6. Patents

G.J., H.R.S, F.V. and J.K. are listed as inventors on the patent application “Use of blocking reagents for reducing unspecific T cell activation”, EP3029067A1, application filed by German Cancer Research Center, DKFZ, University of Tuebingen.

## Data Availability

The data presented in this study are available on request from the corresponding author. The data are not publicly available due to privacy restrictions of voluntary donors.

## Supplementary Materials

The following are available online at https://www.mdpi.com/article/10.3390/cancers13184596/s1, Figure S1: Off-target T cell activation by different PSMAxCD3 bsAb, Figure S2: Expression of PSMA and CD19 on SBC, Figure S3: Off-target T cell activation by bsAb in the presence of the SBC SKW6.4, Figure S4: CD11a/CD18 competition and antigen shift assays, Figure S5: Exemplary gating strategy for flow cytometry-based assays, Figure S6: CD18/CD54 blockade combination.
