# Supplementary material for: CD18 Antibody Application Blocks Unwanted Off-Target T Cell Activation Caused by Bispecific Antibodies
Source: Cancers (Basel). 2021 Sep 13;13(18):4596. doi: 10.3390/cancers13184596 (PMC8467378; doi:10.3390/cancers13184596)
Supplement: Supplementary file 1 [file cancers-13-04596-s001.zip › cancers-1330976-supplementary.pdf]

Supplementary Fig. 1

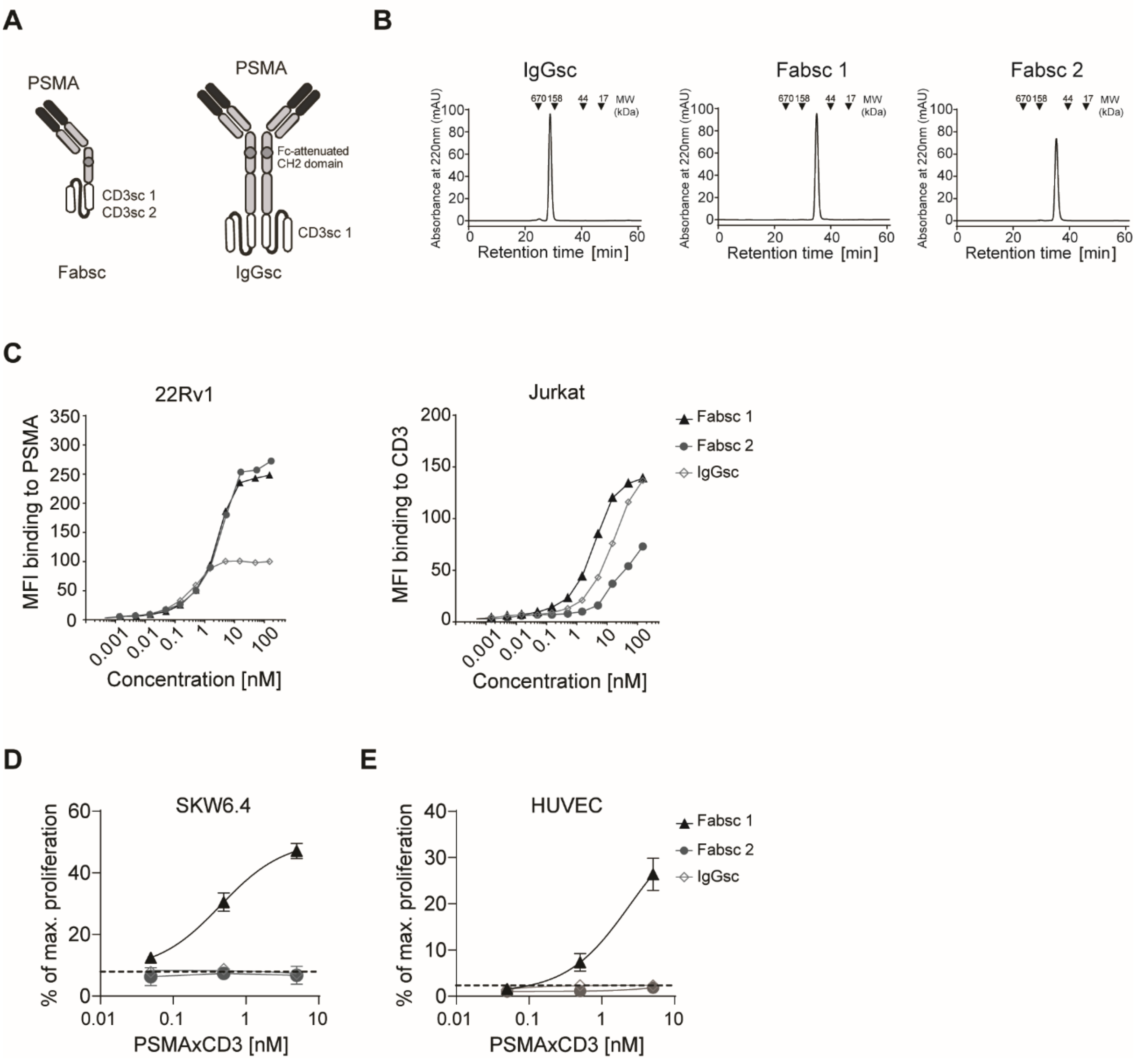

Supplementary Fig. 1 Off-target T cell activation by different PSMAxCD3 bsAb.

(**A**) Schematic representation of two different bsAb formats: Fabsc and IgGsc. Mutations were introduced to the CH_2 domain in order to prevent FcR and complement binding (Fc attenuation). (**B**) Representative gel-filtration profiles of the IgGsc (PSMAX CD3) and Fabsc proteins, containing UCHT-1(Fabsc 1) or OKT-3 (Fabsc 2) as a single chain (**C**) Binding of PSMAxCD3 antibodies in different formats to $PSMA^+$ 22Rv1 cells (left panel) and to $CD3^+$ Jurkat cells (right panel). (**D**) Off-target T cell proliferation in a 3 day ^{3}H-thymidine incorporation assay with PSMAxCD3 antibodies in different formats in the presence of 100,000 PBMC and 100,000 SKW6.4. (**E**) Off-target T cell proliferation in a thymidine assay with PSMAxCD3 antibodies in different formats in the presence of 100,000 PBMC and 50,000 HUVECs.

Supplementary Fig. 2

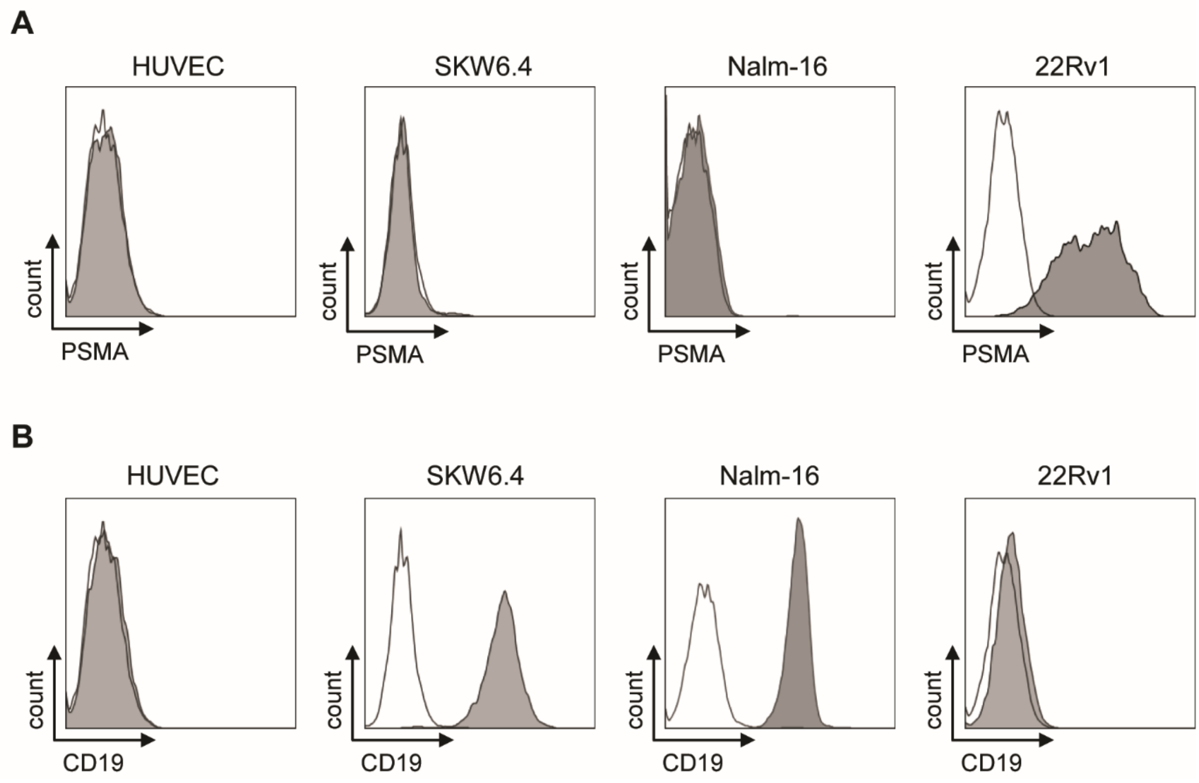

Supplementary Fig. 2 Expression of PSMA and CD19 on SBC.

PSMAxCD3 (**A**) and CD19xCD3 (**B**) expression on different stimulating and non-stimulating cell lines was measured by flow cytometry (n=3, representative results shown). 22Rv1 cells serve as positive control for PSMA expression. Shaded peaks indicate specific binding of both constructs, open peaks indicate the signal obtained with an isotype control.

Supplementary Fig. 3

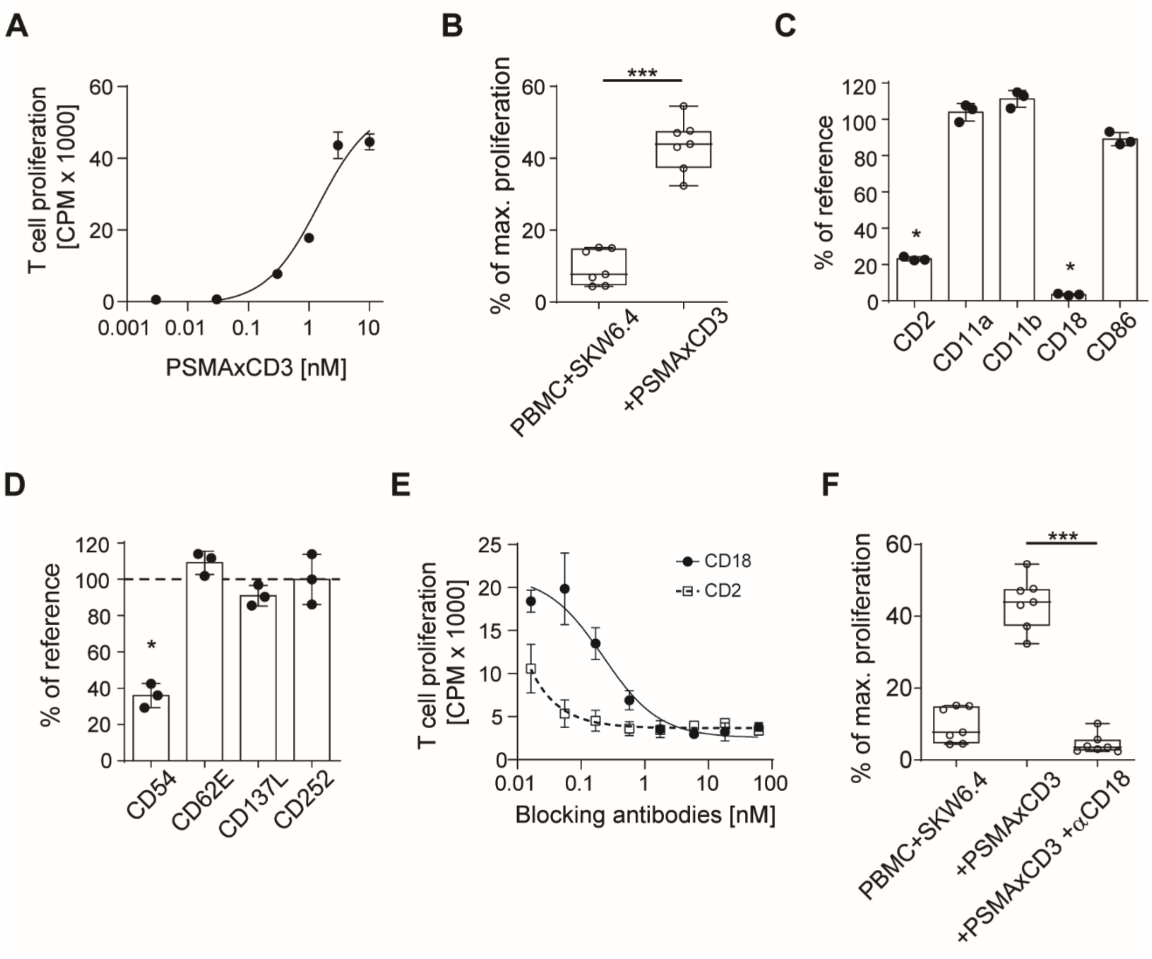

Supplementary Fig. 3 Off-target T cell activation by bsAb in the presence of the SBC SKW6.4.

(**A**) Off-target T cell proliferation in a 3 day ^{3}H-thymidine incorporation assay with a PSMAxCD3 bsAb in the presence of 100,000 PBMC and 100,000 SKW6.4. Representative results from experiments with PBMC from 5 different donors (mean± SD of triplicates). (**B**) 3 day thymidine assay as described in a (n=7) with a PSMAxCD3 bsAb at 5 nM (mean± SD, unpaired t-test). (**C**) Thymidine assay (n=3) using SKW6.4 and a PSMAxCD3 antibody at 5 nM. Addition of different blocking antibodies directed against costimulatory molecules on T cells (mean± SD, unpaired t-test). (**D**) Inhibition of off-target activation by different blocking antibodies directed against costimulatory/adhesion molecules on SBCs in a thymidine assay with a PSMAxCD3 bsAb at 5 nM (n=3, mean± SD, unpaired t-test). (**E**) Inhibition of off-target activation by CD18 and CD2 antibodies in a thymidine assay as described in **C** (n=3, mean± SD, unpaired t-test). (**F**) Inhibition of off-target activation by blocking antibodies against CD18 (n=7, boxplots with min/max whiskers, unpaired t-test) as analyzed by thymidine assays as described in **C.** *p < 0.05, **p < 0.01, ***p < 0.001.

Supplementary Fig. 4

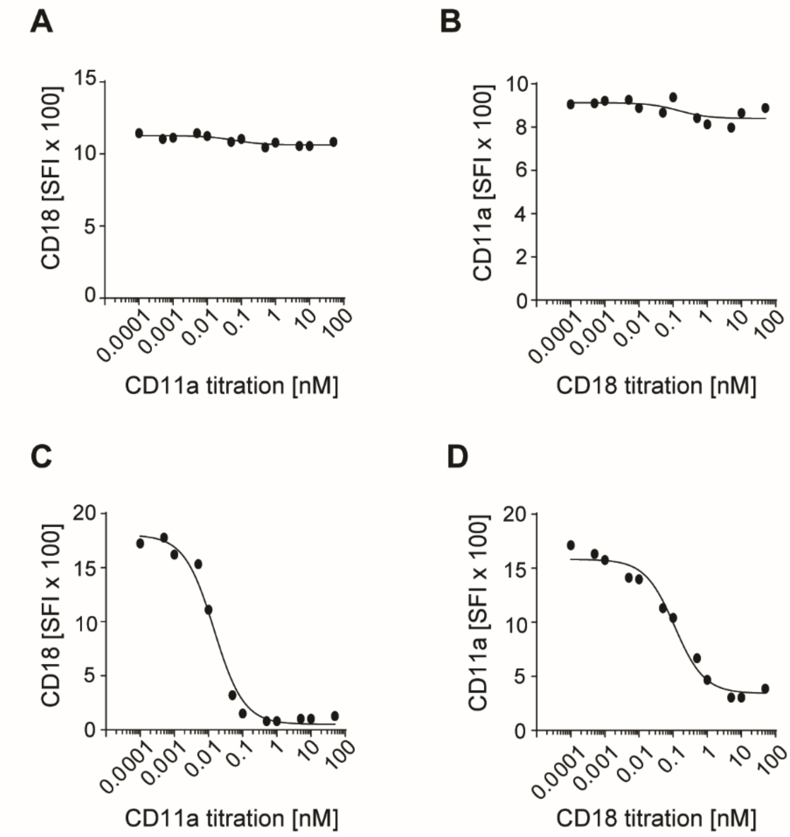

Supplementary Fig. 4 CD11a/CD18 competition and antigen shift assays.

On-target activated T cells (n=6 donors, mean values), in the presence of Nalm-16 and CD19xCD3 bsAb were used for a competition and antigen shift assays. (**A-B**) A competition assay was performed, during which CD11a (**A**) or CD18 (**B**) antibodies were titrated and incubated for 1 hour at 4°C. Subsequently, cells were incubated for an additional hour with 10 µg/ml of directly labeled CD18-PE (**A**) or CD11a-PE (**B**). The results were analyzed by flow cytometry. (**C-D**) Antigen shift assay was performed using on-target activated T cells, which were incubated for 30 hours at 37°C with ascending concentrations of CD11a (**C**) or CD18 (**D**) antibodies. Saturating amounts of directly labeled CD18-PE (10 µg/ml) antibodies (**C**) or unconjugated CD18 antibodies (**D**) were added for one hour at 4°C. All results were obtained by flow cytometry.

Supplementary Fig. 5

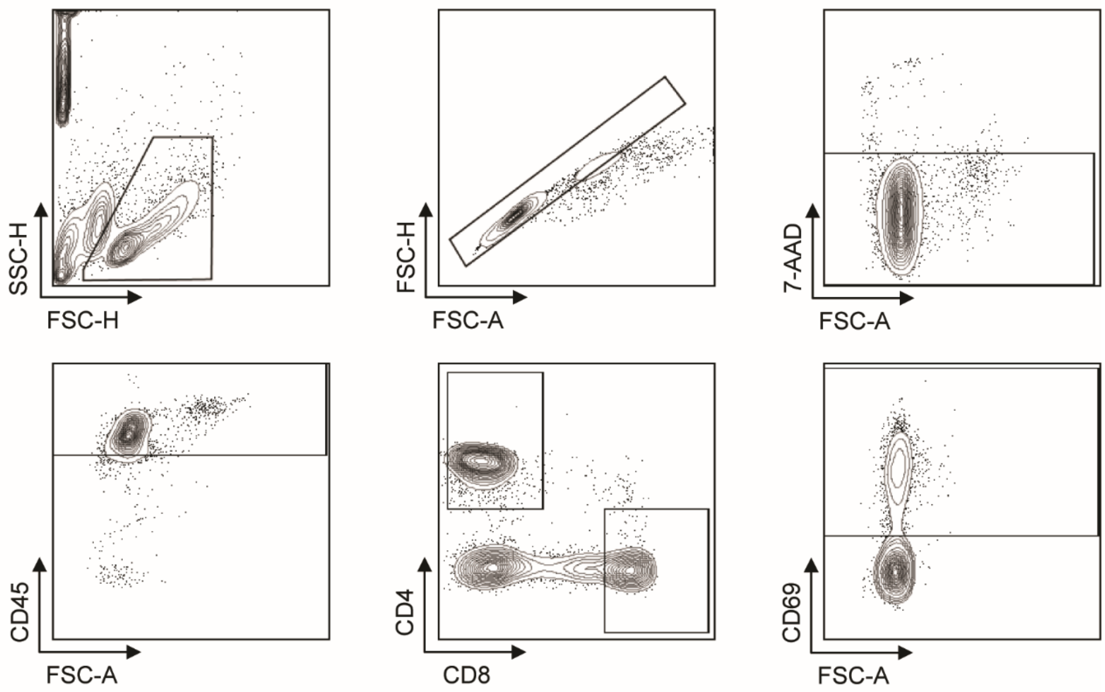

Supplementary Fig. 5 Exemplary gating strategy for flow cytometry-based assays.

(**A**) Exemplary gating strategy and representative data set for flow cytometry-based assays: Mononuclear cells, singlets, viable (7-AAD$^-$), CD45$^+$, CD4$^+$ or CD8$^+$ T cells, CD69$^+$ activated T cells.

Supplementary Fig. 6

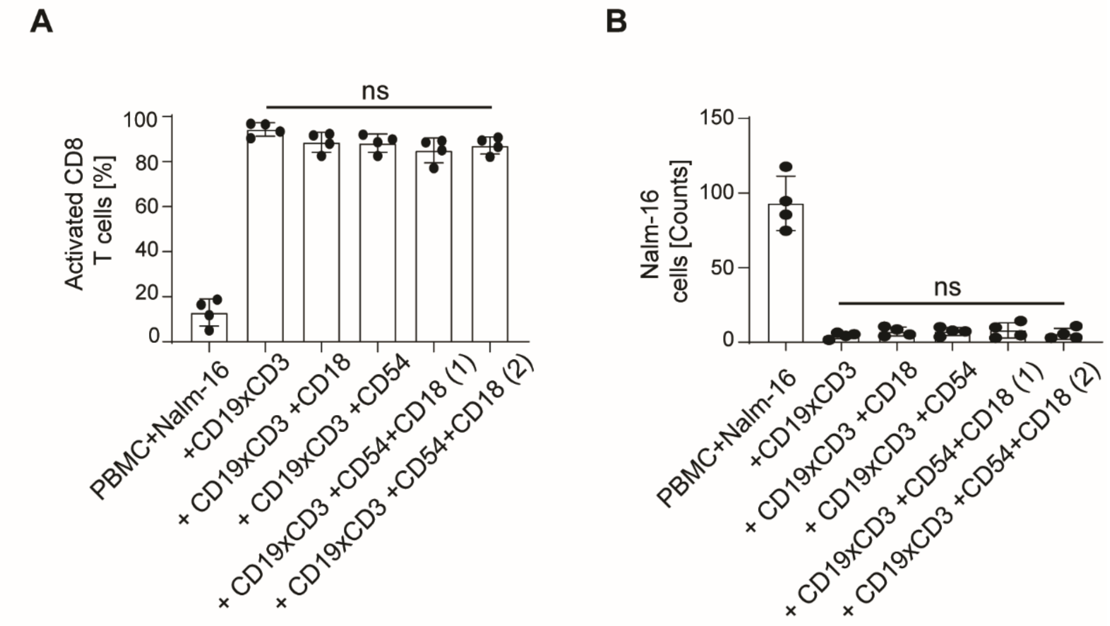

Supplementary Fig. 6 CD18/CD54 blockade combination.

Nalm-16 cells and PBMCs from healthy donors (n=4 donors, mean values), were incubated in the presence of CD19xCD3 bsAb at a final concentration of 5nM. CD18 (1µg/ml), CD54(1µg/ml) antibody alone or in a combination with CD18 blockade at 0.1µg/ml (1) or 0.01µg/ml (2) were also added. On day 3, CD8 T cell activation (**A**) and Nalm-16 cell kill (**B**) were analyzed by flow cytometry. Statistical analyses were performed using unpaired t-test (n=4, mean± SD). ns : non-significant.
